# Crosslinking of Chitosan with Dialdehyde Chitosan as a New Approach for Biomedical Applications

**DOI:** 10.3390/ma13153413

**Published:** 2020-08-03

**Authors:** Katarzyna Wegrzynowska-Drzymalska, Patrycja Grebicka, Dariusz T. Mlynarczyk, Dorota Chelminiak-Dudkiewicz, Halina Kaczmarek, Tomasz Goslinski, Marta Ziegler-Borowska

**Affiliations:** 1 Faculty of Chemistry, Nicolaus Copernicus University in Torun, Gagarina 7, 87100 Torun, Poland; kasiawd@doktorant.umk.pl (K.W.-D.); patrycja.grebicka@vp.pl (P.G.); dudkiewicz.dor@gmail.com (D.C.-D.); halina@umk.pl (H.K.); 2 Chair and Department of Chemical Technology of Drugs, Poznan University of Medical Sciences, Grunwaldzka 6, 60780 Poznan, Poland; mlynarczykd@ump.edu.pl (D.T.M.); tomasz.goslinski@ump.edu.pl (T.G.)

**Keywords:** dialdehyde chitosan, dialdehyde starch, cross-linking, chitosan, Microtox^®^ test

## Abstract

Materials based on natural high molecular compounds are particularly interesting for biomedical applications. It is known that the cross-linking agent used for preparation of biomacromolecule-based materials is as important as used biopolymer. Therefore, natural cross-linkers containing reactive carbonyl groups are of great interest especially for modifying properties of natural polysaccharides. One of the most popular cross-linking agents is glutaraldehyde. Nevertheless, the unreacted particles can be released from the cross-linked material and cause cytotoxic effects. This can be eliminated when using a cross-linker based e.g., on polysaccharides. This article describes quick and efficient synthesis of dialdehyde chitosan (DACS) and its application for the preparation of chitosan films. Materials obtained with different amount of DACS were fully characterized in terms of structure and surface morphology. Thermal and mechanical properties as well as hydrophilic character were also examined. The results obtained were compared with the materials obtained by cross-linking chitosan with low molecular weight glutaraldehyde and high molecular weight cross-linking agent based on polysaccharide–dialdehyde starch. Toxicity of all obtained materials was tested using the Microtox^®^ test. It has been shown that due to better mechanical, thermal and surface properties as well as lower toxicity, dialdehyde chitosan is a very promising crosslinking agent.

## 1. Introduction

Recently, a growing interest in low-toxic materials derived from natural sources or waste has been observed in many fields of material science and applications. Attempts are being made to obtain completely new materials or to modify already known components to improve their properties. In recent years, interest in polysaccharides and materials based on these biopolymers has increased significantly [1,2]. Apart from biocompatibility and biodegradability, polysaccharides are susceptible to chemical modification, which allows their structure and properties to be tailored into the form required for their further use [3,4]. This approach can be seen in many fields of science and, in particular, in the design and synthesis of materials for medicine, pharmacy, and biological applications [5,6,7].

Chitosan, a polysaccharide derived from the partial deacetylation of chitin, is a cationic linear biopolymer [8,9]. It has been widely considered as one of the safest biomaterials for various biomedical applications [10,11,12]. Chitosan-based materials have been studied so far as drug delivery systems [13,14], and wound dressings [15,16]. Also, these materials have been used for scaffold manufacturing in tissue engineering [17,18,19,20], and as dietary supplements in the body mass reduction as well as the cholesterol-lowering formulations [21,22]. Furthermore, materials based on chitosan and their modified derivatives were found to be effective against viruses, including the SARS-CoV-2 that causes COVID-19 coronavirus disease, responsible for the global pandemic outbreak in 2020, which began in late 2019 in China [23].

The presence of reactive functional groups, such as hydroxyl or amino groups in the chitosan structure, allows for fairly easy chemical modification of this biopolymer [24,25]. In order to improve the mechanical and physicochemical properties of chitosan-based materials, it is necessary to cross-link this polysaccharide [6]. Due to the presence of the aforementioned functional groups, cross-linking can occur either by ionic interactions, covalent bonding, or hydrogen bridges. To date, the most common cross-linking agents used for fabrication of chitosan materials are: formaldehyde [26], glutaraldehyde [27], various epoxy compounds [28], glyoxal [29], diethyl squarate [30], pyromellitic dianhydride [31], genipin [32], quinone [33], diisocyanate [34,35], polyethylene glycol (PEG) [36,37], and dialdehyde starch (DAS) [38]. Low molecular cross-linkers, especially still commonly applied glutaraldehyde (Glu), are known to enhance the mechanical properties of the final material successfully, but they are also considered highly toxic. Glutaraldehyde, due to its low molecular weight, can enter the cells of a living organism through several pathways [39]. The awareness of the potential adverse effects of the synthetic low molecular weight cross-linking agents stimulated rapid studies on their natural substitutes [40]. In this regard, the most promising for biomedical applications, due to the lack of toxicity, is dialdehyde starch, which is widely used as a drug carrier in the pharmaceutical industry or in tissue engineering in the preparation of cell carrier hydrogels [41,42,43].

**Figure 1:**
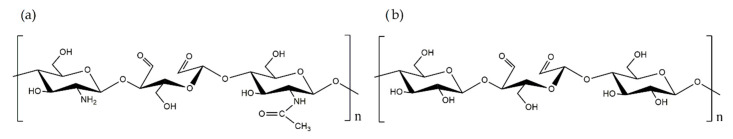
Chemical structure of (**a**) dialdehyde chitosan (DACS) and (**b**) dialdehyde starch (DAS).

Although the structure of dialdehyde chitosan (DACS) [44,45], which is similar to dialdehyde starch in its properties, is already known, there are only two reports available in the literature on the potential utility of this polysaccharide for cross-linking of collagen [46,47]. Dialdehyde chitosan, like dialdehyde starch, contains in its structure reactive carbonyl groups obtained by oxidation of the origin polysaccharide (Figure 1). The mechanism of cross-linking of chitosan after this modification is almost identical to the cross-linking process with the low molecular weight dialdehydes or dialdehyde starch. It can be expected that chitosan materials cross-linked in this way will be characterized by low toxicity while maintaining chitosan properties such as hydrophilicity, which is essential for biomedical applications.

Given the above information, the primary purpose of this work was to obtain chitosan films cross-linked with dialdehyde chitosan, determination of their characteristics, and comparison with materials cross-linked with dialdehyde starch and a small molecule cross-linking agent, which is the commonly used glutaraldehyde. In order to perform the detailed characterization of all received materials, commonly available methods were applied, e.g., scanning electron microscopy (SEM), attenuated total reflection Fourier-transform infrared (ATR-FTIR) spectroscopy, and atomic force microscopy (AFM). Also, the mechanical properties of the obtained films were determined, including their hydrophilic nature using contact angle measurement. The acute toxicity of the prepared chitosan films was preliminarily studied using the Microtox^®^ test.

## 2. Materials and Methods

### 2.1. Materials

Chitosan (low molecular weight: MW = 50 kDa, deacetylation degree = 75–85 %, CAS Number 9012-76-4,), corn starch, sodium periodate, glutaraldehyde, diiodomethane (pure for analysis), glycerol were obtained from Sigma-Aldrich (St. Louis, MO, USA) and used without further purification. Acetic acid, sodium hydroxide, concentrated hydrochloric acid (35%), acetone, and phosphate buffer saline (PBS, pH = 7.4) were purchased from Avantor Performance Materials, (Gliwice, Poland). All solutions were prepared with deionized water.

### 2.2. Dialdehyde Starch Synthesis

The synthesis and characterization of dialdehyde starch was described in detail in our previous work [48].

Briefly, corn starch (1.5 g) was dissolved in deionized water (30 mL). Periodate aqueous solutions of varying concentration (weight ratio of oxidant/corn starch = 0.5, 0.7, 0.9, 1.0, and 1.1) were prepared and then added to the starch suspension under magnetic stirring. The mixture was heated to 40 °C, and stirring was continued in the dark for 3 h. After cooling to room temperature, the appropriate quantity of acetone was poured into the reaction mixture solution to precipitate the product. The white precipitate was collected, washed with deionized water, and dried at room temperature for 24 h. The dialdehyde starch obtained was used as a cross-linking agent for chitosan films.

### 2.3. Dialdehyde Chitosan Synthesis 

Chitosan (1.0 g) was dissolved in acetic acid solution (C = 1% m/m, 100 mL) and mechanically stirred at room temperature for 2 h. Different amounts of sodium periodate were dissolved in deionized water (weight ratio of oxidant/chitosan = 0.5, 0.7, 0.9, and 1.0) and added to the chitosan solution under magnetic stirring. The mixture was heated to 40 °C, and stirring was continued in the dark for 3 h. After cooling to room temperature, the appropriate quantity of acetone was added. The white precipitate was isolated by filtration and washed three times with deionized water. Finally, DACS was dried at room temperature for 24 h.

### 2.4. Cross-Linked Chitosan Films Preparation

Chitosan (0.5 g) was dissolved in acetic acid (C = 1% m/m, 50 mL). Then, the 5%, 10%, or 15% (weight percent based on the dry weight of the polysaccharide) of the appropriate cross-linking agent (dialdehyde starch, dialdehyde chitosan, or glutaraldehyde) was added. The blends were mixed for 2 h with magnetic stirring and were poured onto the leveled glass plates to allow the solvent to evaporate.

### 2.5. Analysis and Characterization

#### 2.5.1. Determination of the Content of Aldehyde Groups (ALD, %)

The number of aldehyde groups by acid-base titration of the modified chitosan solutions was determined [49]. The content of the chitosan units containing the dialdehyde groups was calculated from the Equation (1):

(1) $$\mathrm{ALD}\;\%=\frac{C_{1}V_{1}\;-\;C_{2}V_{2}}{m/M}\times 100\%\;\left(\%\right)$$

where: *C*_1_ (mol/L) and *V*_1_ (dm^3^) are the concentration and volume of NaOH solution, *C*_2_ (mol/L) and *V*_2_ (dm^3^) are the concentration and volume of HCl solution, respectively; *m*—mass of the sample (g), *M*—molecular weight of the repeated unit in dialdehyde chitosan (*M* = 160 g/mol).

DACS (0.1 g) and NaOH solution (5 mL, 0.25 M) were added to the Erlenmeyer flask and heated in 70 °C (water bath) until the sample was dissolved. After cooling, the HCl solution (7.5 mL, 0.25 M) and distilled water (15 mL) were added. Then phenolphthalein solution was added, and the sample was titrated with NaOH solution (0.25 M). The procedure was performed in triplicate for each sample.

#### 2.5.2. Attenuated Total Reflectance Fourier Transform Infrared (ATR-FTIR) Spectroscopy

The ATR-FTIR spectra of chitosan, dialdehyde chitosan, and chitosan cross-linked films were obtained using the Spectrum Two^TM^ spectrophotometer (Perkin Elmer, Waltham, MA, USA) equipped with an ATR accessory with an incorporated diamond crystal. Spectra were recorded from 4000 to 400 cm^−1^ with the resolution of 4 cm^−1^ and 32-scans at room temperature.

#### 2.5.3. Scanning Electron Microscopy (SEM)

The morphology for CS, DACS, films, and cross-sections of films were observed with the use of the 1430 VP model scanning electron microscope manufactured by LEO Electron Microscopy Ltd, Cambridge, England. The films were first immersed in liquid nitrogen to fracture and to obtain clear cross-sections. All samples for which the surfaces and cross-sections were tested were covered with gold.

#### 2.5.4. Contact Angle Measurement

The contact angle of two liquids, glycerol, and diiodomethane, on the surface of chitosan, dialdehyde chitosan and cross-linked films was measured at constant temperature (24 °C) using a goniometer equipped with the system of drop-shape analysis (DSA produced by KRÜSS GmbH, Hamburg, Germany). Each θ is an average of at least 5 measurements. The surface free energy was calculated by the Owens–Wendt method [50].

#### 2.5.5. Thermal Analysis

Thermogravimetric analysis of chitosan, dialdehyde chitosan, and cross-linked films was performed on a SDT 2960 Simultaneous DSC-TGA thermogravimetric analyzer (TA Instruments, New Castle, DE, USA). The measurements were performed at a 10 °C/min heating rate in the range from 20 °C to 600 °C in the atmosphere of nitrogen.

#### 2.5.6. Mechanical Properties

The mechanical properties of films were studied using EZ-Test E2-LX Shimadzu texture analyzer (Shimadzu, Kyoto, Japan). Five samples of each type were cut and tested. The films were placed in the measuring holders and subjected to stretching at the speed of 20 mm/min. The tensile strength and Young’s modulus (E) of each film were measured.

#### 2.5.7. Atomic Force Microscopy (AFM)

Morphological appearance of obtained films was evaluated by the atomic force microscopy (AFM) technique, with MultiMode Nanoscope IIIa Veeco Metrology Inc., Santa Barbara, CA, USA. Roughness parameters such as the root mean square (R_q_), arithmetic mean (R_a_), and R_max_ were calculated for 5 μm × 5 μm scanned area using NanoScope Analysis software, version–1.40, Bruker, Billerica, MA, USA.

#### 2.5.8. Swelling Ability

The swelling ability (SA) was measured by the conventional gravimetric method [51]. The dry sample was weighed and placed in 0.05 M PBS (pH 7.4) at room temperature. After the appropriate incubation time, the films were removed from PBS, placed on absorbent paper, and the wet material was weighted. The value was an average of three measurements. The swelling ability was assessed according to the following Equation (2):

(2) $$\mathrm{SA}=\frac{W_{s}\;–\;W_{d}}{W_{d}}\times 100\%\;\left(\%\right)$$

where *W_s_* and *W_d_* denote the weights of swollen and dry samples, respectively.

#### 2.5.9. Toxicity Assessment

Toxicity of prepared materials was evaluated by applying the materials to Microtox^®^ acute toxicity test, the 81.9% Screening Test, which was performed using Microtox^®^ M500 equipment (Modern Water, Cambridge, UK) with a slight modification [52,53]. To evaluate the toxicities of the films precooled diluent (supplied by the producer) was added to the cuvettes containing *Aliivibrio fischeri* bacteria suspension, which was followed immediately by immersing of the film fragment. The decrease of cell viability was measured with Microtox^®^ M500 and Microtox Omni software (version–4.2, Modern Water, Londyn, UK) based on the cell bioluminescence at 490 nm [54].

## 3. Results and Discussion

### 3.1. Synthesis of Dialdehyde Chitosan and Its Oxidation Degree

The method of oxidation of chitosan to its dialdehyde derivative was described and discussed in the literature [44]. Usual methods of chitosan oxidation with sodium periodate yield a 49% content of aldehyde groups after a 48 h reaction. Only Keshk et al. reported that the reaction of chitosan with potassium periodate within 48 h led to the oxidized polysaccharide with 58.8% content of aldehyde groups [45]. In this work, we decided to elaborate our previously reported method for the oxidation of starch for dialdehyde chitosan synthesis [4,48]. In this method, reaction time was only 3 h. After determining the number of aldehyde groups in the sugar unit for dialdehyde chitosan, the ratio of reagents 1:1 was chosen (Table 1). As shown in Table 1 these conditions allowed obtaining chitosan with the highest content of aldehyde groups—58%.

**Figure 2:**
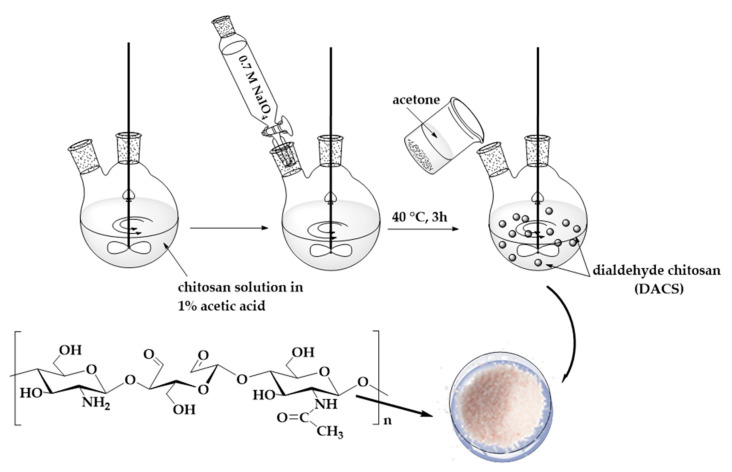
Scheme of the synthesis of dialdehyde chitosan.

The applied method compared to previous work describing DACS synthesis by Keshl et al. leads to oxidized chitosan with the same oxidation degree after a 16-fold reduction of reaction time [45]. The scheme for the synthesis of dialdehyde chitosan is presented in Figure 2. It should be pointed out that the reaction procedure and the work up are not demanding, as the dialdehyde product is precipitating from the reaction mixture after the addition of acetone.

**Figure 3:**
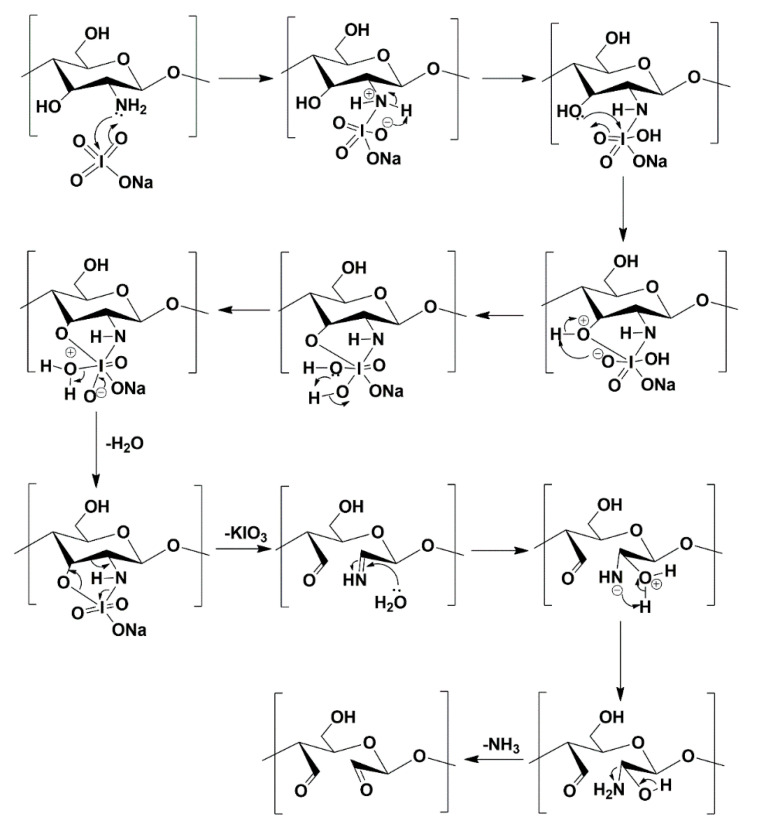
Oxidation of chitosan to dialdehyde chitosan (DACS) with sodium periodate.

The adapted reaction is a one-step oxidation with sodium periodate as an oxidation agent. The mechanism for periodate oxidation of chitosan was already reported by Keshk et al. (Figure 3) [45]. It factors in the ammonia formed during the reaction, which was detected and quantified. As a result of this reaction, dialdehyde chitosan is obtained in the form of a light beige amorphous powder, which is soluble in water.

### 3.2. Characterization of Dialdehyde Chitosan

In order to define the structure, morphology and thermal stability of prepared dialdehyde chitosan, standard techniques were applied: infrared spectroscopy, microscopic analysis, contact angle measurements, thermal analysis, and X-ray diffraction (XRD).

#### 3.2.1. ATR-FTIR Spectroscopy

**Figure 4:**
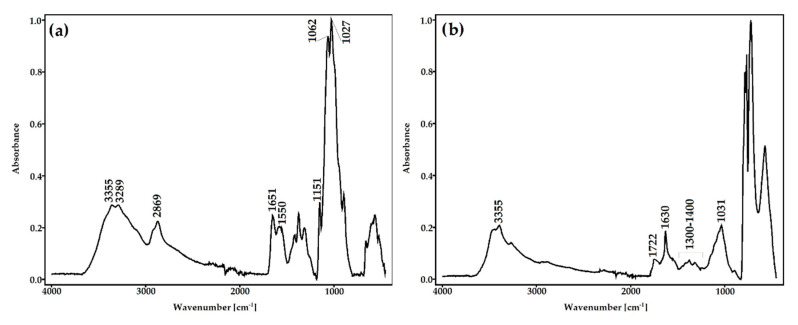
Attenuated total reflectance Fourier transform infrared (ATR-FTIR) spectra of (**a**) chitosan and (**b**) dialdehyde chitosan.

In the spectrum of unmodified chitosan, the characteristic absorption bands at 3355, 3289 cm^−1^ (attributed to O–H and N–H stretching vibrations), 2869 cm^−1^ (CH stretching), 1651cm^−1^ (C=O stretching of amide I), 1550 cm^−1^ (N–H bending of amide II), 1151 cm^−1^ (C–O–C stretching), and 1062, 1027 cm^−1^ (C–O stretching) were observed (Figure 4a). The obtained spectrum is consistent with the literature data [55,56].

After oxidation, a new band at 1722 cm^−1^ representing the carbonyl stretching vibrations appeared (Figure 4b). Additionally, a newly formed sharp band at 1630 cm^−1^ derived from carbonyl group confirmed oxidation of chitosan. The changes in the shape and intensity of the hydroxyl band (3355 cm^−1^) can be explained by the opening of cyclic structure and oxidation of chitosan saccharide units. Moreover, the intensity of absorption bands in DACS, attributed to pyranose rings, decrease significantly. Considerable changes were also observed in the vibration range of the C–O–C groups (1300–1400 cm^−1^), which proves the effective modification of glucoside rings caused by their opening and oxidation in positions C2 and C3.

#### 3.2.2. Scanning Electron Microscopy (SEM)

**Figure 5:**
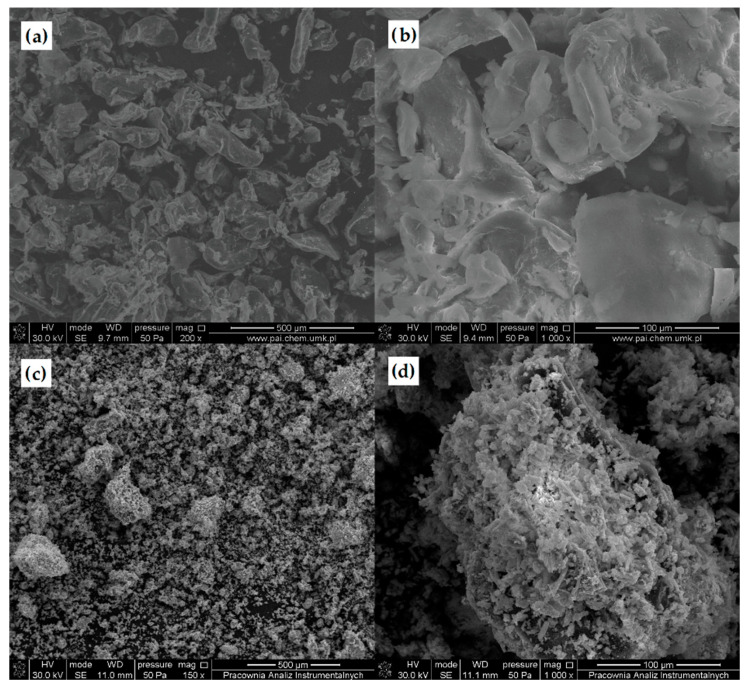
Scanning electron microscopy (SEM) pictures of (**a**,**b**) chitosan, and (**c**,**d**) dialdehyde chitosan powder.

The morphology of unmodified CS and DACS was studied using scanning electron microscopy (Figure 5). The SEM images of native chitosan powder exhibited loosely bound grains with irregular and rough surfaces with the size ranging from 100 to 350 µm [57]. After oxidation, the irregular grains of chitosan disappeared and were transformed into rugged shapes with lots of needle forms. The length of the needle forms was less than 100 µm, and the average size of coarse shapes was between 300–400 µm.

#### 3.2.3. Contact Angle Measurement

The results of contact angle measurements obtained for the chitosan and dialdehyde chitosan films are presented in Table 2. As can be seen, the contact angle for chitosan with the polar liquid (glycerin) is higher than for the non-polar diiodomethane. This means that both samples are less wetted by the polar glycerin. It is confirmed by higher (γ_s_^d^) than the polar component (γ_s_^p^). Therefore, the results confirm the hydrophobic nature of the sample, which are comparable with literature reports [58]. Over two-times higher value of polar component indicate a greater polarity of DACS when compared to the native polysaccharide. This may be because the aldehyde groups in the structure of the oxidized chitosan align towards the surface of the polymer film, and therefore, dialdehyde chitosan is more hydrophilic than unmodified chitosan. Similar conclusions were reported in the literature for oxidized cellulose [59]. This is especially beneficial for biomedical applications of this material and may result in its easier penetration of biological membranes and better solubility in body fluids.

#### 3.2.4. Thermal Analysis

**Figure 6:**
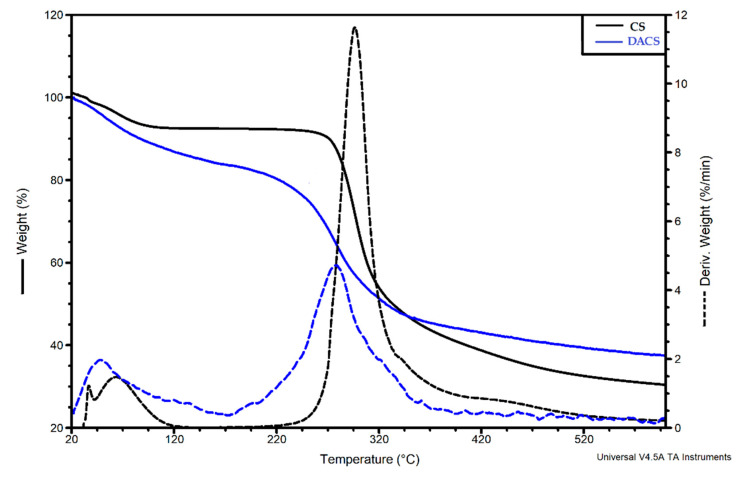
The thermogravimetric analysis–difference thermogravimetry (TGA-DTG) curves of chitosan and dialdehyde chitosan.

To determine the thermal properties of dialdehyde chitosan, a TGA-DTG (thermogravimetric analysis–difference thermogravimetry) analysis was performed. Thermograms for chitosan and dialdehyde chitosan are shown in Figure 6. For unmodified chitosan, the first step of degradation is observed between 20 and 120 °C, with a 7% mass loss assigned to the evaporation of the adsorbed water. The main decomposition stage is observed between 220 and 530 °C and is associated with the 62% mass loss corresponding to the thermal degradation of the chitosan polymer chain. The TG curve of DACS also shows two degradation stages, which are consistent with the literature data [60]. These steps can be attributed to the same degradation processes as for pure chitosan. The temperature at the maximum decomposition rate T_max_ = 281 °C in dialdehyde chitosan is slightly lower than for unmodified chitosan (T_max_ = 295 °C. This may be due to the fact that in the structure of chitosan dialdehyde, the pyranose rings have already been opened by the oxidation reaction.

#### 3.2.5. X-ray Diffraction (XRD)

**Figure 7:**
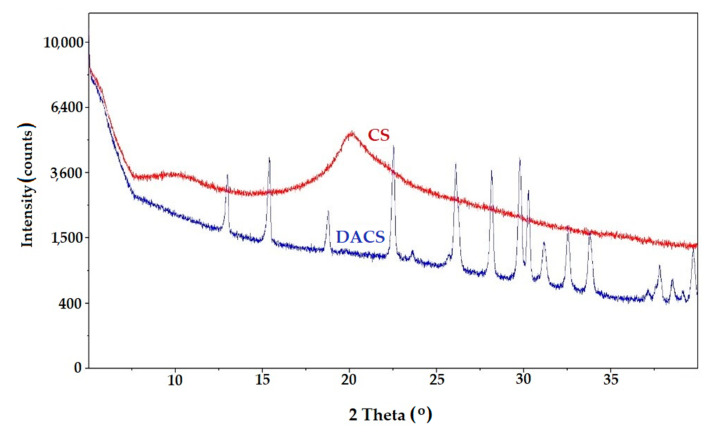
The X-ray diffraction (XRD) patterns of chitosan (CS) and dialdehyde chitosan (DACS).

The XRD patterns of chitosan and dialdehyde chitosan are shown in Figure 7. Chitosan is a semi-crystalline polysaccharide with two main crystalline peaks at 2θ = 10.3 and 20.1°, which corresponds well with the literature reports [61,62]. In literature, Miller indices (hkl) of diffraction peaks were (0 2 0) and (1 1 0), respectively [63]. Signals in this X-ray diffraction pattern are assigned to crystalline form I and II, respectively. This is as a result of two inter- and intramolecular H-bonding among the functional groups present on the saccharide units. However, the observed signals are very wide, with a high broad halo indicating a relatively low degree of macromolecular ordering, which is typical for natural polysaccharides. The disappearance of the signal at 2θ about 20° in the XRD pattern of DACS proves the complete disappearance of the crystalline phase characteristic for chitosan [44]. Many narrow intensive signals proving the presence of a crystalline product were observed in the DACS diffractogram. It should be added that despite careful, repeated washing of the reaction product (DAS), it is not possible to completely remove the excess NaIO_4_ oxidant and probably its reduced product (NaIO_3_), which tends to complexation of polysaccharide molecules. A similar situation was noted in the case of dialdehyde starch obtained by the sodium periodate oxidation reaction. It is expected that, when sodium periodate is used as an oxidizer to polysaccharides, a stable iodate complex is formed with the dialdehyde product [48]. Strong interactions between the dialdehyde chitosan and the oxidant (IO_3_^−^) result in the formation of the complex and are present as signals in the diffractogram.

### 3.3. Formation of Chitosan Films by Cross-Linking with Various Cross-Linkers

As previously stated, the main purpose of this work was to obtain new materials based on chitosan cross-linked with a dialdehyde derivative of this polysaccharide. Due to the fact that chitosan materials are widely described and very widely studied, in particular in medical sciences, the obtained films were compared with materials cross-linked with glutaraldehyde and dialdehyde starch. In order to prepare film samples, the cross-linking agent was used in three different amounts based on the weight of chitosan 5%, 10%, and 15% of the total.

Obtained materials were characterized in order to define the structure, morphology, thermal stability, mechanical properties, degree of swell, and toxicity. All of the tested properties affect the utility of the materials in biomedical applications. It is expected that the best material for these functions will have adequate hydrophilicity, strength, and porosity, which will allow free transfer in body fluids, interaction with biological membranes, and free growth of cells on the material surface. Due to their intended use, it is also essential that the materials are not toxic.

#### 3.3.1. ATR-FTIR Spectroscopy

**Figure 8:**
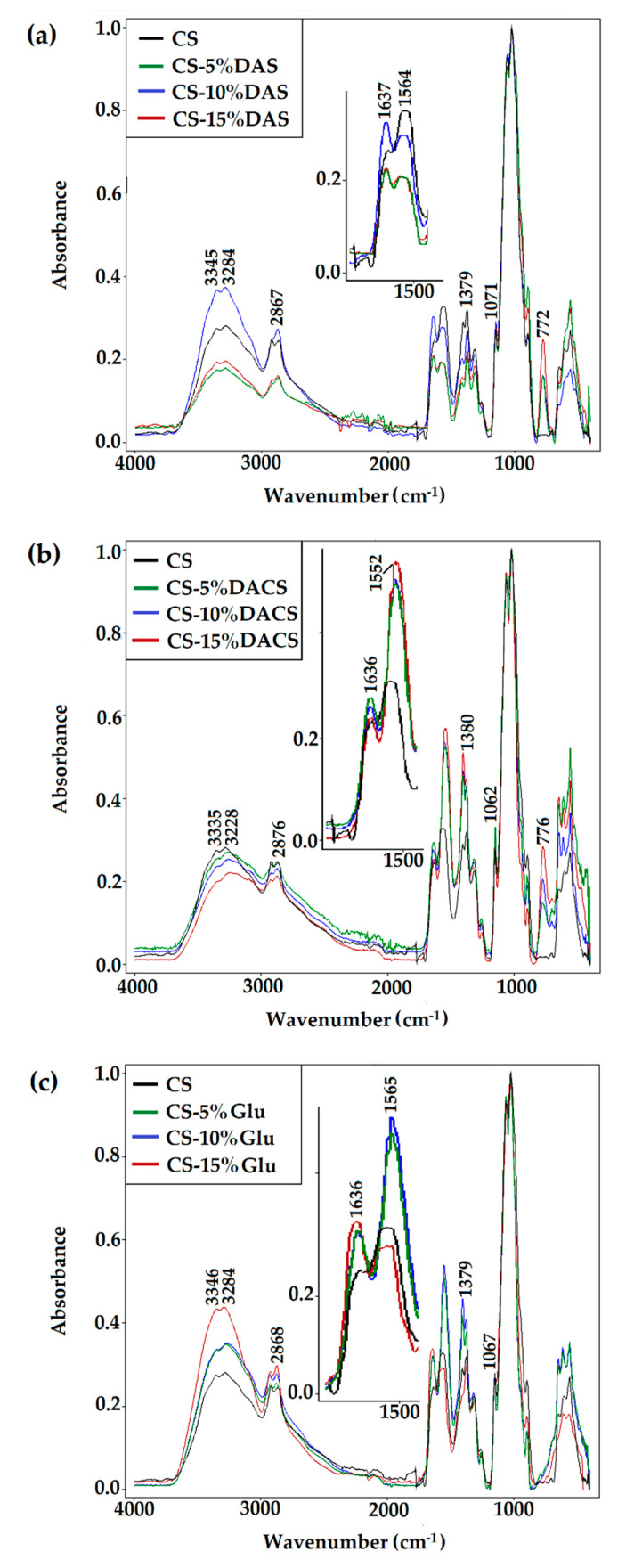
ATR-FTIR spectra of chitosan films cross-linked by (**a**) dialdehyde starch (DAS), (**b**) dialdehyde chitosan (DACS), and (**c**) glutaraldehyde (Glu).

The analysis of the shape and position of characteristic bands supplied information about molecular structures and conformational changes that follow the cross-linking process. The ATR-FTIR spectra of cross-linked chitosan materials are presented in Figure 8, and the data are collected in Table 3.

After chitosan cross-linking with DAS, DACS, or Glu, some changes in the position and shape of the bands were observed in all spectra. Cross-linking of chitosan with dialdehyde compounds, regardless of the cross-linking agent’s molecular weight, occurs by forming an imine bond (Schiff base formation) between the cross-linker carbonyl group and the amino group of the chitosan. In all samples, the band at 1636 cm^−1^, a characteristic absorption peak of imine groups, confirmed this reaction [64]. Moreover, in chitosan films cross-linked with macromolecular cross-linkers, DAS, and DACS, a new band near 770 cm^−1^ is observed. It may be assigned to the =C–H bond (aldehyde group of cross-linker not linked to chitosan) deformation stretching vibrations.

To summarize, in all of the ATR-FTIR spectra of prepared materials, regardless of the cross-linking agent and its ratio, there is explicit confirmation that the chemical cross-linking reaction with the dialdehyde agent was successful.

#### 3.3.2. Scanning Electron Microscopy (SEM)

**Figure 9:**
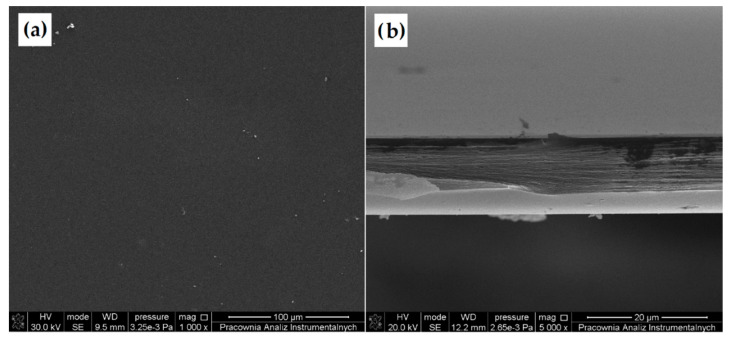
SEM images of (**a**) chitosan surface and (**b**) cross-section of chitosan film.

**Figure 10:**
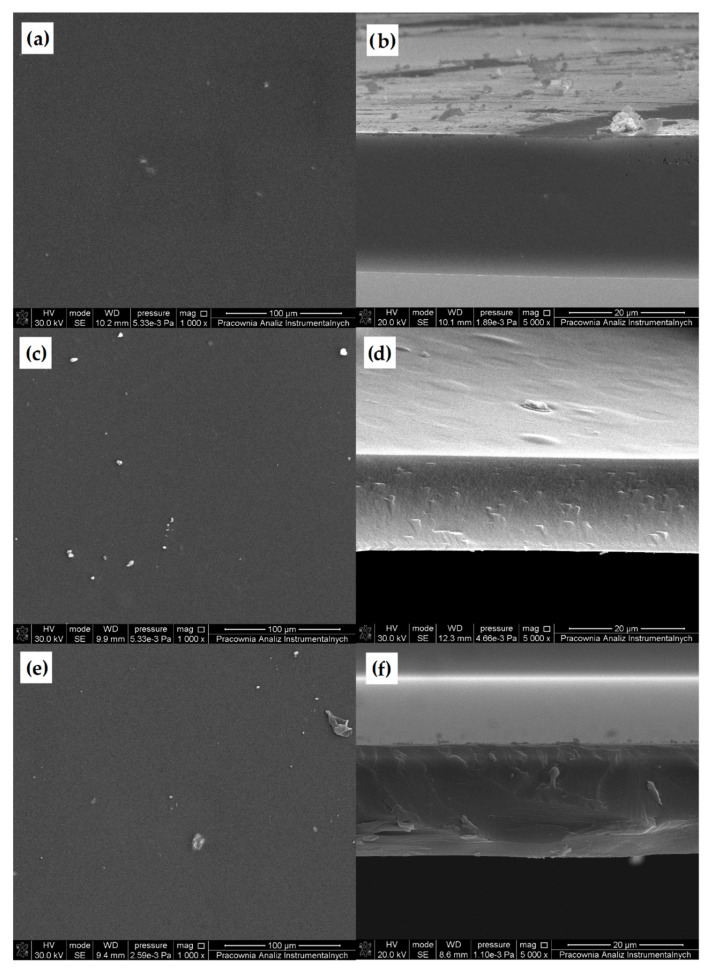
SEM images of chitosan films cross-linked by 5% (**a**) DACS, (**c**) DAS, and (**e**) Glu, and SEM images of the cross-section of chitosan films cross-linked by 5% (**b**) DACS, (**d**) DAS, and (**f**) Glu.

Scanning electron microscopy was applied to determine the surface morphology of prepared samples. Moreover, for better material characteristics of the obtained films, a cross-section was also taken (Figure 9 and Figure 10). The SEM images of all of the prepared samples are presented in Supplementary Materials (Figures S1 and S2).

Almost in all cases, regardless of the cross-linking agent and the quantity used, a flat and smooth film surface can be observed. This indicates that prepared chitosan films are homogeneous. Films cross-linked with 10%, and 15% of dialdehyde starch are exceptions (Figure S1c,d). The surfaces of these films are significantly more folded and contain numerous nodules compared with the surface of pure chitosan and films with a different cross-linker and DAS in the amount of 5% by mass. The aggregates may be larger to the gel formation caused by the higher cross-linker content, resulting in a more significant surface heterogeneity. The analysis of a cross-section of all of the prepared cross-linked chitosan materials indicated that the films are also homogeneous in bulk. This confirmed that the process occurs in the whole material volume for all cross-linkers. Also, based on the selection of materials, which are cross-linked with 10% and 15% addition of dialdehyde starch, it is known that the heterogeneity applies only to the surface, whereas the material is homogeneous deeper under the surface.

#### 3.3.3. Atomic Force Microscopy (AFM)

**Figure 11:**
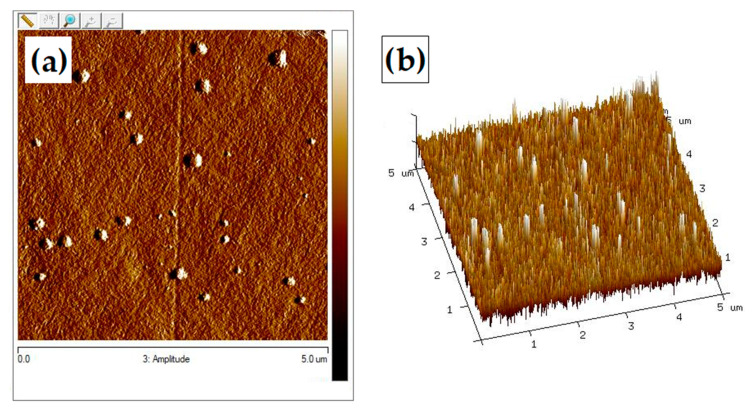
Atomic force microscopy (AFM) images of chitosan in (**a**) 2D scale and (**b**) 3D scale.

**Figure 12:**
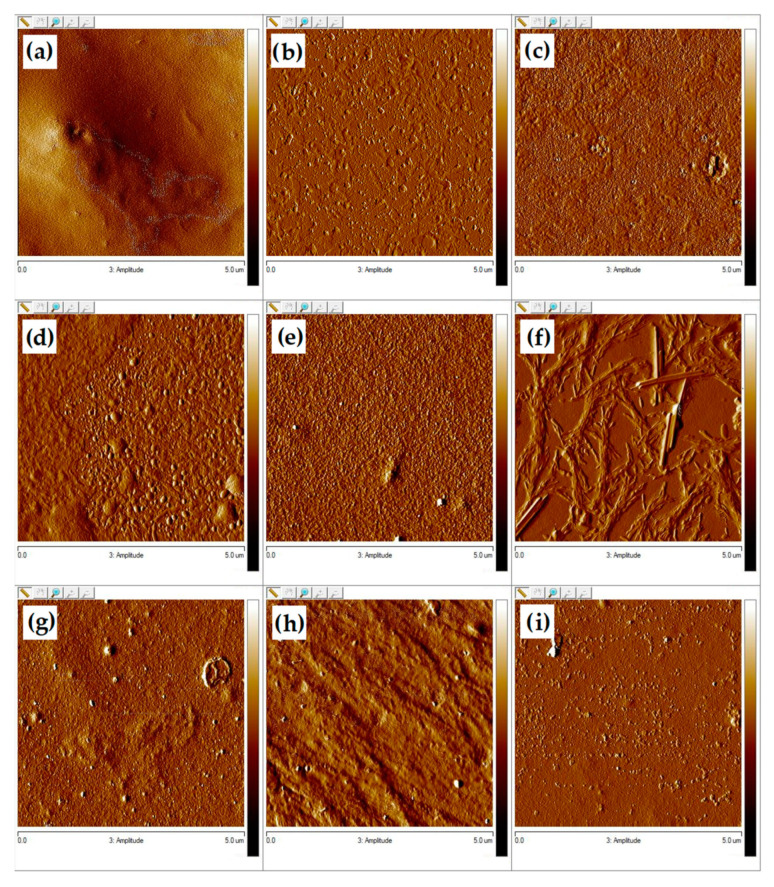
AFM images in 2D scale of chitosan films cross-linked by (**a**) 5%, (**b**) 10%, (**c**) 15% DACS, (**d**) 5%, (**e**) 10%, (**f**) 15% DAS, and (**g**) 5%, (**h**) 10%, (**i**) 15% Glu.

**Figure 13:**
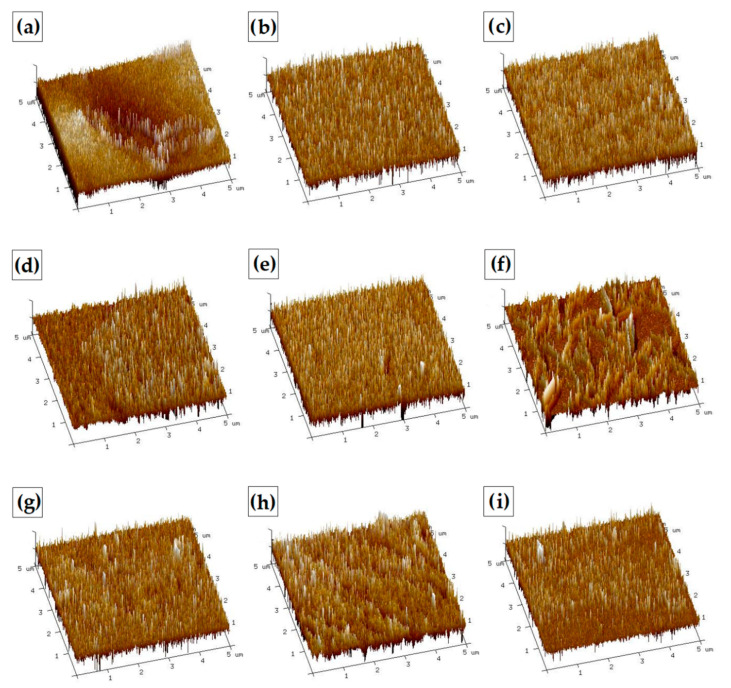
AFM images in 3D scale of chitosan films cross-linked by (**a**) 5%, (**b**) 10%, (**c**) 15% DACS, (**d**) 5%, (**e**) 10%, (**f**) 15% DAS, and (**g**) 5%, (**h**) 10%, (**i**) 15% Glu.

The surface roughness is of great importance in later material applications. The topography of the surface of the prepared sample was evaluated by atomic force microscopy. One of the primary advantages of rough materials for biomedical applications is the ability to increase the proliferation of connective tissue cells on their surface. Therefore, for applications, such as regenerative medicine or tissue engineering, material roughness is a particularly desired feature. The images in 3D and 2D scales are presented in Figure 11, Figure 12 and Figure 13. As can be seen, the surface morphology of chitosan films without cross-linking is significantly different from cross-linked materials. For pure chitosan, a relatively smooth morphology was observed, which is mainly related to the physical properties of the chitosan such as high intrinsic chain stiffness [65]. The surface of chitosan films cross-linked with dialdehyde polysaccharides is rather rough; however, for CS-Glu, a significant decrease in roughness is observed (Table 4).

As can be seen from the data in Table 4, the material with the highest roughness is chitosan cross-linked with 15% addition of dialdehyde starch. The R_max_ value for this sample is more than twice higher than for pure chitosan. A promising material due to surface roughness is also chitosan cross-linked with 5 and 10% addition of dialdehyde chitosan with R_max_ values of 132 and 119, respectively. The roughness results obtained confirmed the observations from SEM.

#### 3.3.4. Contact Angle Measurement

In order to assess the hydrophilic-hydrophobic nature of the obtained materials, measurements of contact angles using glycerin (polar) and diiodomethane (non-polar) were performed. The results of these measurements, as well as the calculated value of surface free energy, the polar and dispersive components, are presented in Table 5.

All the values of glycerin average contact angle were less than 90°, which proves that the materials are rather hydrophilic. Moreover, it is worth noting that all of the cross-linked material surfaces are characterized by better wettability by glycerol than non cross-linked chitosan (Table 5). Pure chitosan has a free surface energy of about 30.70 mJ/m^2^ [66]. As shown in Table 5, the values of polar and dispersive components of surface free energy depend on the type of used cross-linker. Chitosan films cross-linked by DAS and DACS were characterized by slightly lower values of surface free energy than the pure chitosan. However, after comparing the surface energy components, it is seen that the share of the polar component is much higher than in the case of pure chitosan and increases with the addition of a cross-linking agent. A comparison of the value of this component and glycerin contact angle shows that cross-linked chitosan films are more polar than pure chitosan and their hydrophilicity increases with the increasing cross-linking agent ratio.

The results obtained for films cross-linked with 10% and 15% of glutaraldehyde are surprising. Both the value of glycerin contact angle and the polar free surface energy component indicate the highest hydrophilic nature of these materials. This is probably related to the fact that it has a low molecular weight compared to polysaccharide cross-linking agents. For molecules with a lower mass, the proportion of the hydrophobic fragment of the structure is smaller. Also, they can more easily “accumulate” at the surface of the material, resulting in increased interaction of functional groups with the solvent.

Based on the results obtained, it can be concluded that all the obtained materials meet the expectations for potential biomedical applications. The values of contact angle and surface free energy polar component allow stating that these materials are promising for such applications.

#### 3.3.5. Thermal Analysis

**Figure 14:**
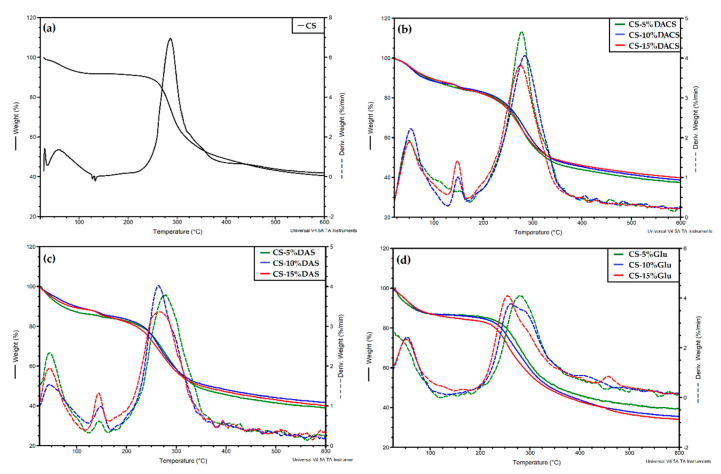
The TGA-DTG curves of (**a**) chitosan, chitosan cross-linked by various amounts of (**b**) DACS, (**c**) DAS, and (**d**) Glu.

For a better characterization of the materials, thermal analysis of the obtained films was performed. The results of the thermogravimetric analysis of chitosan and chitosan cross-linked films are presented in Figure 14 and Table 6.

As can be seen on the thermogravimetric curves and based on the data collected in Table 6, pure chitosan film exhibits two thermal degradation stages [67,68,69]. The first stage (between 29 °C and 140 °C) is characterized by approximately 8% loss of the initial weight, and is related to the evaporation of water. The intensive decomposition of chitosan takes place in the second stage, in the temperature range of 180–420 °C, with 51% weight loss. This probably results from the detachment of side hydroxyl groups, main chain scission, and pyranose ring-opening reactions.

In thermograms of chitosan films cross-linked with DACS and DAS three degradation steps were observed. The exception is the sample CS-5% DACS, where only two stages of decomposition can be found, similar to pure chitosan film. It is probably the result of the similarity of polymer chain structures of chitosan and cross-linker. For materials cross-linked with low molecular weight glutaraldehyde, two decomposition steps were also observed.

The first decomposition stage, for all cross-linked films, ranges between 50 and 120 °C and shows about 9–14% loss in weight, due to the loss of bounded and adsorbed water similar to unmodified polysaccharide. The next step of thermal degradation of all cross-linked materials is the intensive destruction, which takes place in the temperature range between 170 and 350 °C and shows about 50% loss in weight due to the degradation of the chitosan chain. As mentioned, for samples cross-linked with glutaraldehyde, only two degradation stages were observed. These stages can be attributed to the same degradation processes as for pure chitosan film. The degradation of the low molecular weight cross-linker occurs in the first stage at a temperature between 20 and 100 °C. The weight loss corresponding to these temperatures is slightly higher than for other materials.

As mentioned, in the case of films cross-linked with dialdehyde polysaccharides, there is an additional stage of decomposition in the temperature range between 120 and 180 °C. The weight loss at this stage is small, at the level of 3–5%, which may indicate that this stage consists in breaking the cross-linking of chitosan films. This step does not appear in the case of material cross-linked with 5% addition of dialdehyde starch. The residue at 600 °C is comparable for all materials.

#### 3.3.6. Mechanical Properties

**Figure 15:**
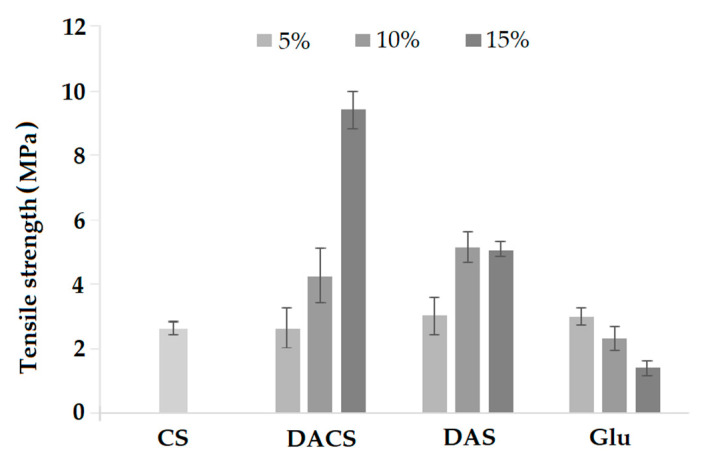
Tensile strength of pure chitosan (CS) and chitosan cross-linked by 5%, 10% and 15% adding of various cross-linkers (DACS, DAS and Glu).

**Figure 16:**
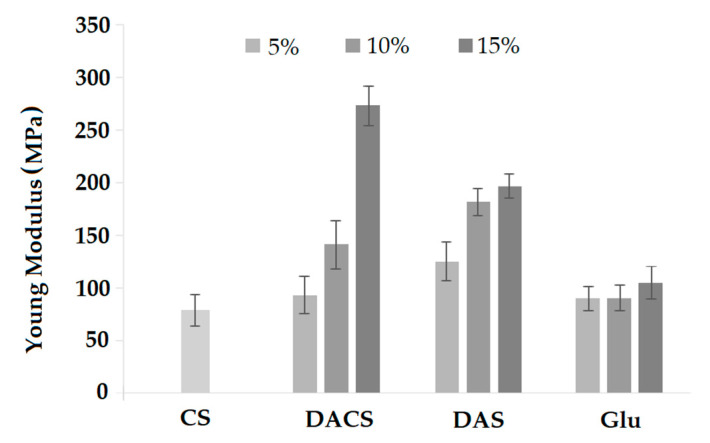
Young’s modulus of pure chitosan (CS) and chitosan cross-linked by 5%, 10% and 15% adding of various cross-linkers (DACS, DAS and Glu).

**Figure 17:**
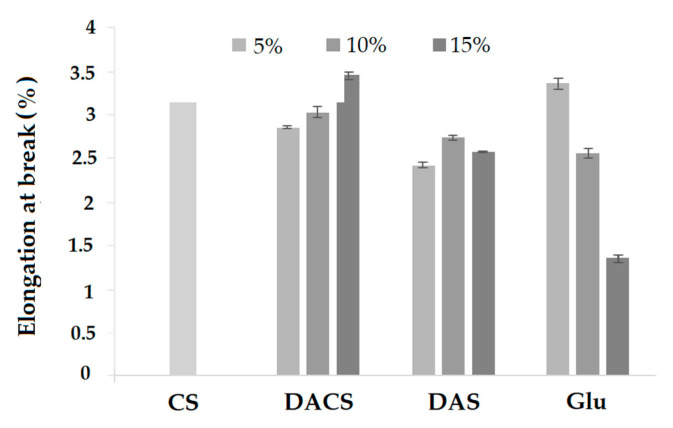
Elongation at break of of pure chitosan (CS) and chitosan cross-linked by 5%, 10% and 15% adding of various cross-linkers (DACS, DAS and Glu).

The mechanical properties of polymer materials indicate their strength and applicability in conditions where they are subjected to mechanical forces. Parameters describing these properties for the materials are tensile strength, Young’s modulus, and elongation at break which are presented in Figure 15, Figure 16 and Figure 17 for all of the prepared samples.

The value of tensile strength for CS sample was about 3 MPa. After cross-linking with an addition of 5% of different cross-linkers, the tensile strength value is practically the same as for pure chitosan. However, tensile strength increases with rising rates of cross-linking agents for chitosan films cross-linked with dialdehyde polysaccharides. The most significant difference in this parameter was observed for CS-15%DACS sample, where the tensile strength increased to 9.43 MPa, which is 3.5 times more than for pure chitosan. An almost two-fold increase in strength value in comparison to pure chitosan was also observed for CS-10%DAS and 15%DAS films. The tensile strength of materials cross-linked with low molecular weight glutaraldehyde shows the opposite trend. This fact is in agreement with the literature data, where the increase of the glutaraldehyde corresponds to the decrease of this parameter up to the value almost two times lower than observed for non-cross-linked chitosan films [70]. Based on the comparison of stress at the break of the prepared materials, it can be concluded that the creation of a new cross-linking network between chitosan and dialdehyde polysaccharides leads to the improvement of mechanical properties [41,71].

Young’s modulus value for pure chitosan film is about 75 MPa, and it is almost the same as for films of chitosan cross-linked with 5% addition of DACS and Glu. For the sample cross-linked with 5% of DAS, the Young’s modulus value is about 125 MPa. In the case of samples cross-linked with glutaraldehyde, no significant changes in the value of the Young modulus are observed with increasing amounts of cross-linking agent. For samples cross-linked with dialdehyde polysaccharides DACS and DAS, along with the increase in the amount of cross-linking agent, an approximately two-fold increase in the Young’s modulus is observed. The highest value of the Young’s modulus is observed for the material cross-linked with a 15% addition of dialdehyde chitosan (3.5 times higher than for pure chitosan).

Elongation at break is an important feature for materials in terms of applications. This parameter is a measure of the tensile ability of a film before break. The percentage value of elongation at break was calculated from the elongation in length and the original length of the films. For pure CS sample it was about 3.35%. In the case of materials cross-linked with dialdehyde chitosan, the percent elongation increases with increasing amount of cross-linking agent. The elongation at break of samples cross-linked with low molecular weight glutaraldehyde shows the opposite trend. The smallest value of the elongation at break is observed for the material cross-linked with a 15% addition of glutaraldehyde (2 times smaller than for pure chitosan).

#### 3.3.7. Swelling Ability

The swelling process depends on the structure of the polymer and the type of solvent. Swelling is essential if we consider the suitability of the material for the preparation of e.g., hydrogels, which is significant for biomedical applications. Cross-linked polymers swell in a limited way because the solvation energy is not large enough to break the newly formed cross-linking (covalent) bonds between the polymer chains, contrary to unmodified linear chitosan. The wettability promotes swelling of the polymer sample because the interactions between macromolecules and solvent molecules facilitate their diffusion into the interior.

**Figure 18:**
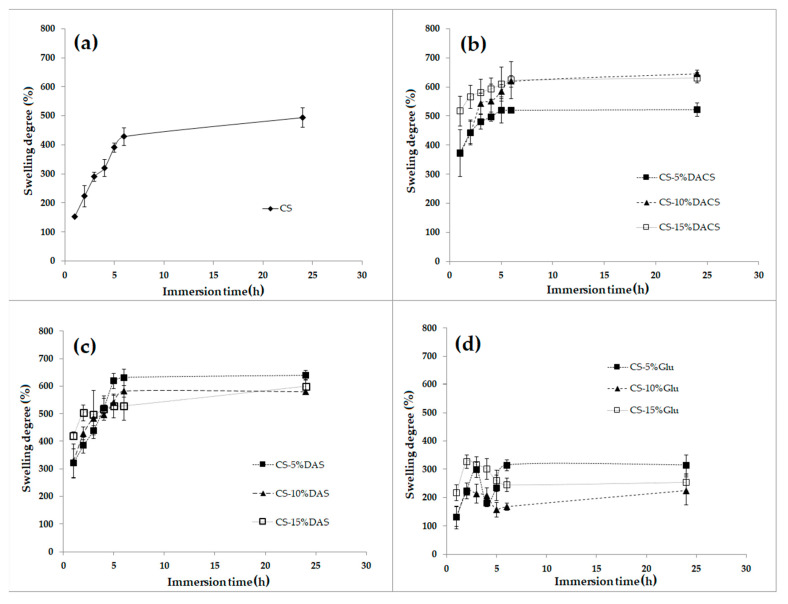
The swelling degree of (**a**) chitosan (CS), chitosan films cross-linked with 5%, 10% and 15% (**b**) dialdehyde chitosan (DACS), (**c**) dialdehyde starch (DAS), and (**d**) glutaraldehyde (Glu).

Chitosan has various functional groups in the structure and is rather easily wetted by polar solvents [72,73], which confirms the value of glycerin contact angle. The swelling behavior of chitosan and chitosan films cross-linked by various cross-linkers measured in PBS (phosphate buffer solution) is shown in Figure 18.

As presented, pure chitosan after the first hour of immersion in PBS has a swelling degree value of 153%. However, this swelling ratio is two times lower than for chitosan films cross-linked with dialdehyde polysaccharides after the same period. The lowest degree of swelling after one hour is observed for materials cross-linked with glutaraldehyde. It can also be seen that as the glutaraldehyde content increases, which is associated with a higher degree of cross-linking, the swelling capacity slightly decreases. Also, it can be seen that for these samples, the degree of swelling decreases with the immersion time. This may further indicate that the samples decompose in the PBS starting from the third immersion hour. DACS and DAS cross-linked films show similar swelling, but the most promising are samples cross-linked with 10% and 15% addition of dialdehyde chitosan—even after 25 h, the samples still show a slight increase in swelling, which means that they did not undergo degradation in the buffer. It is very constructive for medical applications and shows that these materials can be successfully used, for example, for the production of hydrogel bandages.

#### 3.3.8. Microtox^®^ Toxicity Study

**Figure 19:**
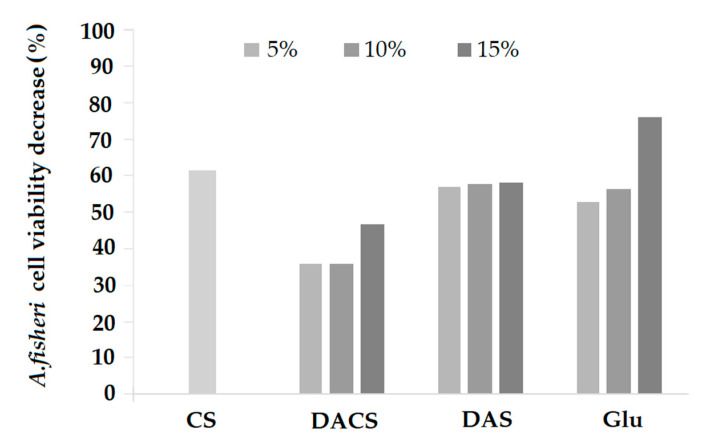
*A. fischeri* cell viability decreases upon exposition to chitosan-based films. CS—non-cross-linked chitosan, DACS—chitosan films cross-linked with dialdehyde chitosan, DAS—chitosan films cross-linked with diacetyl starch, and Glu—chitosan films cross-linked with glutaraldehyde. The colors represent the content of the cross-linking agent (5%, 10%, 15%) in the film.

The Microtox^®^ toxicity test is based on the measurement of the bioluminescence of the Gram-negative *A. fischeri* bacteria, which is correlated to the bacterial metabolism and cell viability [52]. The toxicity of pure chitosan (CS) suspension was also tested. The obtained values of a percentage reduction in the survival of *A. fischeri* bacteria are shown in Figure 19.

For pure chitosan, the cell viability decrease of *A. fischeri* cells was equal to 61%. Such high value is most probably caused by the antibacterial activity of this biopolymer [64,74], as the material itself is non-toxic [75]. Survival decreases for chitosan films cross-linked by DACS were about 20% lower than for pure chitosan film when DACS was used in 5% or 10% concentration. The decrease of cell viability could be correlated with the increase of the DACS content, as 15% of DACS in the films resulted in 53% cell viability, which was still less toxic than CS. A different relationship was observed for chitosan films cross-linked with DAS. The observed decrease in cell viabilities was not influenced by the concentration of DAS (57%, 58% and 58% cell viability decrease for 5%, 10%, and 15% DAS concentration, respectively). Additionally, it seems that cross-linking with DAS had almost no effect on the toxicity of the chitosan films. However, the use of glutaraldehyde as the cross-linking agent caused a reduction in viability at higher concentrations (24% cell viability for 15% Glu content), which may be related to the toxicity of glutaraldehyde [76]. The Microtox^®^ 81.9% toxicity test has also recently been used in similar study by Sharma et al. [77]. They researched the cell toxicity of the chitosan-based nanohydrogel nanocomposite, fabricated by facile sol-gel polymerization technique, chitosan-cl-poly(AAc)/ZrPO_4_. The acute toxicity (EC_50_) values of chitosan-cl-poly(AAc)/ZrPO_4_ at 257 and 121 mg/mL towards *A. fischeri* after 15 and 30 min of incubation indicated that the material obtained is non-toxic and has a biocompatible nature.

In order to understand the results obtained, it is necessary to analyze them in context of chitosan’s antimicrobial activity. The antimicrobial properties of chitosan and its mode of action were broadly reviewed by Kong et al. [78]. Its antimicrobial action is affected by four factors. The first factor is microbial and is related to microorganism and cell age. The second one is intrinsic and is bound to chitosan’s positive charge, molecular weight, concentration, and chelating properties, whereas the third one is physical and is related to its water-solubility and solid state. The final factor, the environmental one, involves the ionic strength in medium, pH, temperature, and time of reaction of this biomolecule. Noteworthy, antimicrobial chitosan activities create further possibilities to use this material in various physical forms, e.g., films, fibers, beads, hydrogels in the food, medical and textile industries. Such antimicrobial polymers can be applied as packaging materials, wound-dressing materials, edible films and coatings and wrapping materials, as well as in tissue engineering and drug delivery. The antimicrobial activity of chitosan, its derivatives and composites against Gram-positive and Gram-negative bacteria has been demonstrated in many studies [79,80,81,82]. It was found that the outer barrier present in Gram-negative bacteria makes these species very susceptible to chitosan [83,84,85]. Moreover, it was also noted that chitosan can pass the cell membrane of Gram-negative bacteria, which could further trigger intracellular responses and its interference with DNA and RNA synthesis [86,87,88]. The morphology of solid chitosan and its derivatives strongly influence its antimicrobial activity which was presented by Takahashia et al. for the powdered chitosan membranes in their action against *Staphylococcus aureus* [89]. In addition, differences in antimicrobial action of chitosan hydrogels towards *Escherichia coli* and *S. aureus* bacteria were observed depending on the immobilization of this material on the surface of composites [90]. Generally, it was presented in many studies that the chitosan’s molecular weight influences biological activity and that chitosan films with higher molecular weights presented no antimicrobial activity [88,91].

In many studies chitosan as a biopolymer was found to be relatively safe and non-toxic towards mammalian cells. The results obtained are a good introduction for further research of chitosan and its derivatives after the treatment with the natural cross-linking agents, as well as prospective biomedical applications.

## 4. Conclusions

In summary, dialdehyde chitosan was obtained by one step and a quick reaction with sodium periodate. The content of carbonyl groups was dependent on the amount of oxidant used for the reaction. The oxidation of chitosan to dialdehyde polysaccharide was confirmed with ATR-FTIR, SEM images and XRD analysis. The strong interactions between the dialdehyde chitosan and the residue of oxidant (IO_3_^−^) as the complex formation was observed to be similar to starch oxidation by this same method. 

The ability of the dialdehyde chitosan obtained to chitosan film cross-link was evaluated and compared with chitosan films cross-linked with dialdehyde starch and glutaraldehyde with cross-linker addition of 5%, 10%, and 15 %. All of the prepared chitosan films are homogeneous except materials cross-linked with 10% and 15% of dialdehyde starch. However, all of materials were homogeneous in depth as confirmed on the cross-section. The surface of all materials was rough. The highest roughness was observed for chitosan cross-linked with 15% addition of dialdehyde starch and 5 and 10% addition of dialdehyde chitosan. The R_max_ value for this samples were more than twice higher than for pure chitosan. The values of contact angle and surface free energy polar component for prepared chitosan films allow to state that due to the nature of the surface, chitosan materials cross-linked with dialdehyde chitosan are promising for biomedical applications. Moreover, modification of chitosan films with dialdehyde polysaccharides results in high swelling ability, probably due to the polar nature of DAS and DACS while the samples cross-linked with glutaraldehyde were degraded in the PBS solution. Based on the comparison of stress at the break of the prepared materials, it can be concluded that creation of new cross-linking network between chitosan and dialdehyde chitosan leads to better mechanical properties than for materials cross-linked with glutaraldehyde.

Microtox^®^ toxicity studies of the obtained materials compared to pure chitosan have shown that the lowest toxicity effect is characteristic for materials obtained by cross-linking chitosan with DACS. These results together with good swelling, mechanical and thermal strength results as well as surface morphology make dialdehyde chitosan a very promising material as a cross-linking agent for biomacromolecules in biomedical applications.

## Figures and Tables

**Table 1:** Degree of oxidation of dialdehyde chitosan, where CS is chitosan, OA is an oxidation agent, and ALD is the content of aldehyde groups.

Sample	CS:OA	ALD, %
DACS_1_	1:0.5	22
DACS_2_	1:0.7	29
DACS_3_	1:0.9	35
DACS_4_	1:1	58

**Table 2:** Surface free energy (γ_s_) and dispersive (γ_s_^d^) and polar (γ_s_^p^) components calculated for the native and the dialdehyde chitosan.

Sample	Average Contact Angle (θ, °)		Surface Free Energy (mJ/m^2^)		
	Measuring Liquid				
	Glycerin	Diiodomethane	γ_s_	γ_s_^d^	γ_s_^p^
CS	82.0	56.0	30.70	27.46	3.23
DACS	70.0	51.8	35.10	27.26	7.84

**Table 3:** The position of main bands (cm^−1^) in ATR-FTIR spectra of CS, CS–DACS, CS–DAS, and CS–Glu films.

Sample	–OH	–NH	C–H	C=N	–NH	C–N	C–O	=C–H
CS	3342	3278	2877	1636	1578	1381	1064	-
CS-DACS	3335	3228	2876	1636	1552	1380	1062	776
CS-DAS	3345	3284	2867	1637	1564	1379	1071	772
CS-Glu	3346	3284	2868	1636	1565	1379	1067	-

**Table 4:** The roughness parameters (R_q_, R_a_, and R_max_) for chitosan (CS) film and chitosan cross-linked by 5%, 10% and 15% adding of various cross-linkers (DACS, DAS and Glu).

Roughness Parameters (nm)	Sample			
	CS	CS-5DACS	CS-10DACS	CS-15DACS
R_q_	9.24	21.9	20.3	5.57
R_a_	7.49	17.0	15.4	4.38
R_max_	89.9	132.0	119.0	56.6
		**CS-5DAS**	**CS-10DAS**	**CS-15DAS**
R_q_	-	13.7	16.1	27.4
R_a_	-	10.8	13.8	21.6
R_max_	-	79.5	87.9	201.0
		**CS-5Glu**	**CS-10Glu**	**CS-15Glu**
R_q_	-	9.4	5.75	2.69
R_a_	-	7.93	4.55	1.64
R_max_	-	46.1	47.7	76.9

**Table 5:** The average contact angle for glycerin and diiodomethane, total surface free energy (γ_s_), its dispersive (γ_s_^d^), and polar (γ_s_^p^) components calculated for films of chitosan (CS) and chitosan cross-linked by 5%, 10% and 15% adding of various cross-linkers (DACS, DAS and Glu).

Sample	Average Contact Angle (θ, °)		Surface Free Energy (mJ/m^2^)		
	Measuring Liquid				
	Glycerin	Diiodomethane	γ_s_	γ_s_^d^	γ_s_^p^
CS	82.0	56.0	30.70	27.46	3.23
CS-5%DACS	76.1	67.8	27.31	18.59	8.73
CS-10%DACS	76.0	73.9	25.72	14.98	10.74
CS-15%DACS	69.3	67.7	30.37	17.33	13.04
CS-5%DAS	80.6	70.0	25.00	18.50	6.84
CS-10%DAS	81.4	64.9	26.70	21.51	5.20
CS-15%DAS	78.3	72.0	25.20	16.50	8.70
CS-5%Glu	72.5	70.3	28.21	16.41	11.80
CS-10%Glu	65.3	67.6	32.51	16.67	15.86
CS-15%Glu	53.7	67.6	39.78	14.78	25.00

**Table 6:** Thermal parameters of pure chitosan (CS), dialdehyde chitosan (DACS), dialdehyde starch (DAS), and chitosan films cross-linked by 5%, 10% and 15% adding of various cross-linkers. (TGA-DTG analysis in nitrogen atmosphere).

Sample	First Stage		Second Stage			Third Stage			Residue 600 °C, (%)
	T_max_ (°C)	Δm (%)	T_o_ (°C)	T_max_ (°C)	Δm (%)	T_o_ (°C)	T_max_ (°C)	Δm (%)	
CS	30, 60	8	-	-	-	145	286	51	40
DACS	52	11	-	-	-	160	281	45	41
DAS	72	10	-	-	-	178	292	70	20
CS-5%DACS	53	13	-	-	-	175	278	49	37
CS-10%DACS	56	12	126	148	3	175	285	46	39
CS-15%DACS	51	10	127	149	5	169	275	44	40
CS-5%DAS	47	13	120	145	-	180	279	47	39
CS-10%DAS	65	10	117	148	4	174	264	44	42
CS-15%DAS	52	9	113	149	5	173	269	45	40
CS-5%Glu	*	13	-	-	-	164	278	47	39
CS-10%Glu	54	12	-	-	-	140	261	51	36
CS-15%Glu	54	14	-	-	-	146	257	52	34

* No maximum on DTG; mass decreases gradually.

## Supplementary Materials

The following are available online at https://www.mdpi.com/1996-1944/13/15/3413/s1, Figure S1: SEM images of chitosan films cross-linked by (a) 10%, (b) 15% DACS, (c) 10%, (d) 15% DAS, and (e) 10%, (f) 15% Glu, Figure S2: SEM images of the cross-section of chitosan films cross-linked by (a) 10%, (b) 15% DACS, (c) 10%, (d) 15% DAS, and (e) 10%, (f) 15% Glu.
