# Supplementary material for: Crosslinking of Chitosan with Dialdehyde Chitosan as a New Approach for Biomedical Applications
Source: Materials (Basel). 2020 Aug 3;13(15):3413. doi: 10.3390/ma13153413 (PMC7435867; doi:10.3390/ma13153413)
Supplement: Supplementary file 1 [file materials-13-03413-s001.pdf]

materials

MDPI

Supplementary Material

Crosslinking of Chitosan with Dialdehyde Chitosan as A New Approach for Biomedical Applications

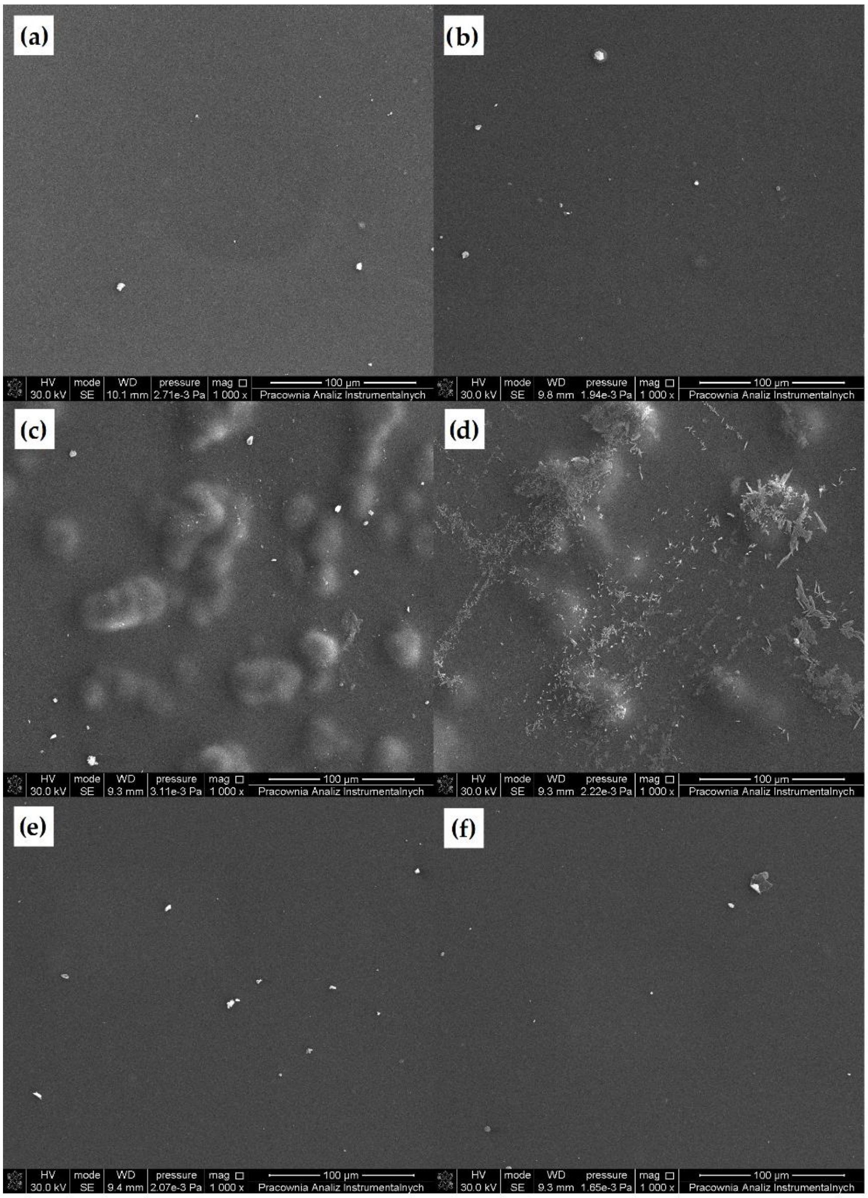

Figure S1. SEM images of chitosan films cross-linked by (**a**) 10%, (**b**) 15% DACS, (**c**) 10%, (**d**) 15% DAS, and (**e**) 10%, (**f**) 15% Glu.

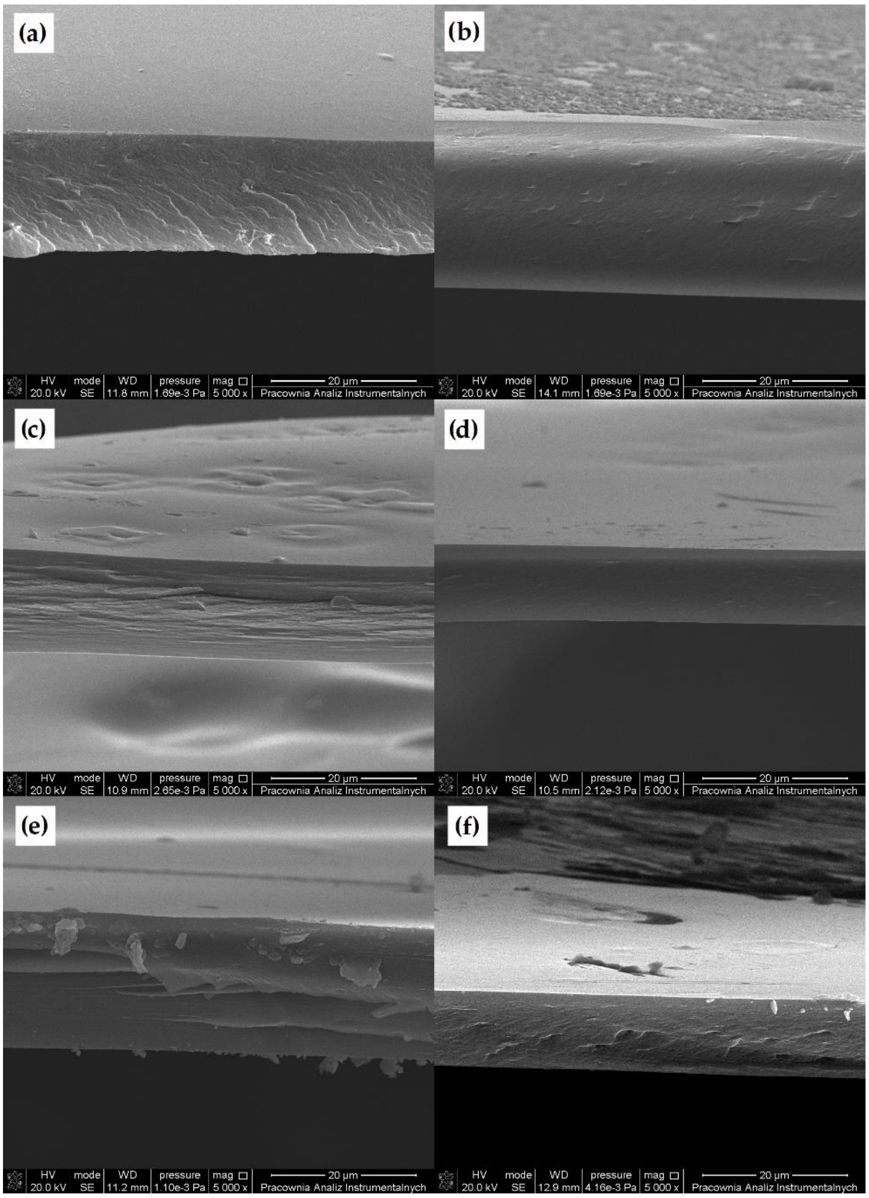

Figure S2. SEM images of the cross-section of chitosan films cross-linked by (**a**) 10%, (**b**) 15% DACS, (**c**) 10%, (**d**) 15% DAS, and (**e**) 10%, (**f**) 15% Glu.

© 2020 by the authors. Submitted for possible open access publication under the terms and conditions of the Creative Commons Attribution (CC BY) license (http://creativecommons.org/licenses/by/4.0/).
